# Supplementary material for: Risk factors for sacrococcygeal pilonidal sinus: a systematic review and meta-analysis supplemented by genetic causal assessment
Source: Front Surg. 2026 Jan 7;12:1718589. doi: 10.3389/fsurg.2025.1718589 (PMC12819706; doi:10.3389/fsurg.2025.1718589)
Supplement: Supplementary file 2 [file Datasheet2.zip › Supplementary Data 2/MR_pipeline_p5e-6/leaveone_plots/prot-a-618.finngen_R12_L12_PILONIDALCYST.pdf]

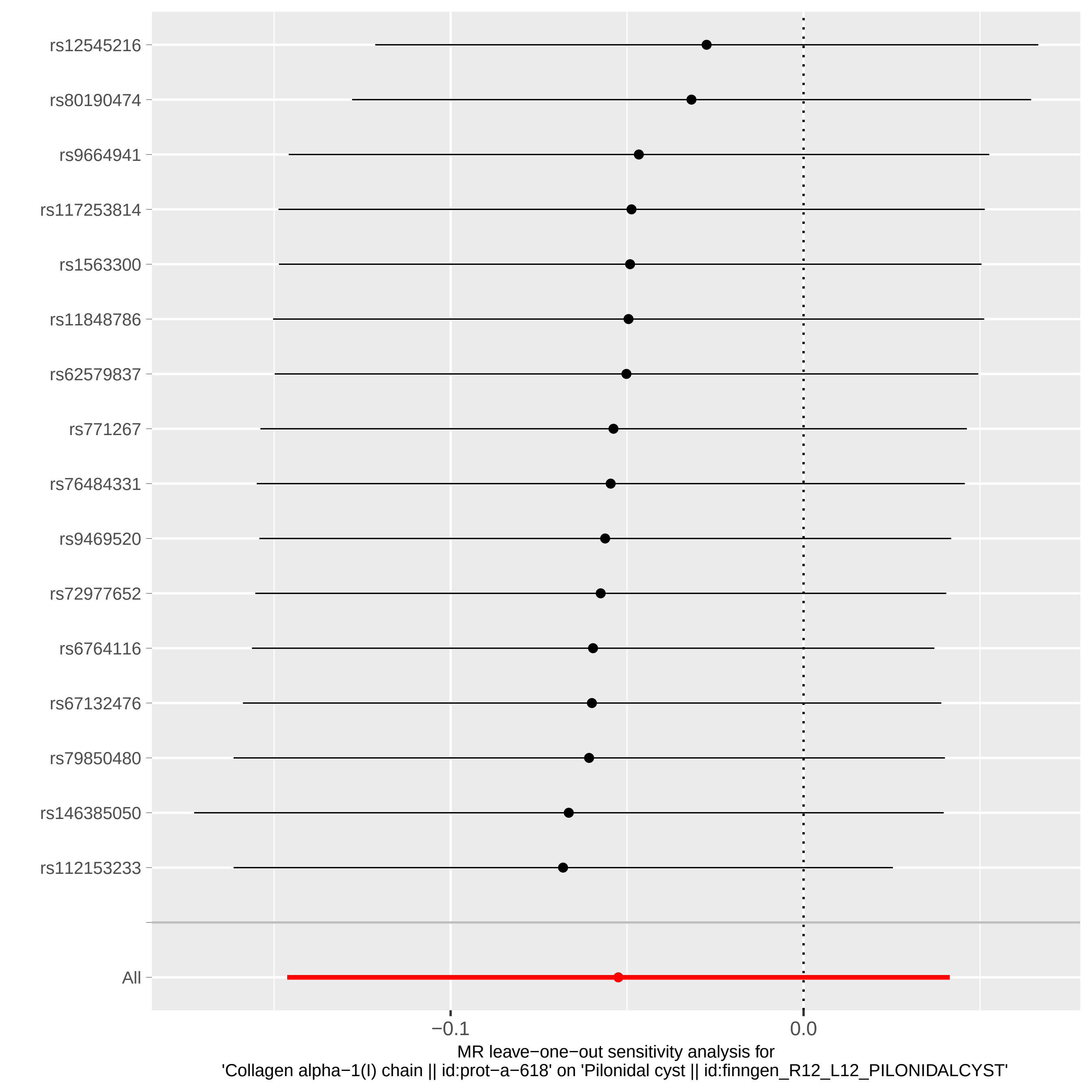

rs12545216
rs80190474
rs9664941
rs117253814
rs1563300
rs11848786
rs62579837
rs771267
rs76484331
rs9469520
rs72977652
rs6764116
rs67132476
rs79850480
rs146385050
rs112153233
All
−0.1
0.0
MR leave−one−out sensitivity analysis for
'Collagen alpha−1(I) chain || id:prot−a−618' on 'Pilonidal cyst || id:finngen_R12_L12_PILONIDALCYST'
